# Biosensors to Monitor Water Quality Utilizing Insect Odorant-Binding Proteins as Detector Elements

**DOI:** 10.3390/bios9020062

**Published:** 2019-05-14

**Authors:** Spiros D. Dimitratos, Allison S. Hommel, Kenneth D. Konrad, Lauren M. Simpson, Jessica J. Wu-Woods, Daniel F. Woods

**Affiliations:** 1 Inscent, Inc., 17905 Sky Park CIR STE P, Irvine, CA 92614, USA; sdimitratos@fullcoll.edu (S.D.D.); allison@inscent.com (A.S.H.); ken@inscent.com (K.D.K.); lauren@inscent.com (L.M.S.); jess@inscent.com (J.J.W.-W.); 2 Department of Biology, Natural Sciences Division, Fullerton College, Fullerton, CA 92832, USA

**Keywords:** biosensor, insect, odorant-binding, protein, coliform, fecal contamination, chemosensory, water

## Abstract

In the developing world, the identification of clean, potable water continues to pose a pervasive challenge, and waterborne diseases due to fecal contamination of water supplies significantly threaten public health. The ability to efficiently monitor local water supplies is key to water safety, yet no low-cost, reliable method exists to detect contamination quickly. We developed an in vitro assay utilizing an odorant-binding protein (OBP), AgamOBP1, from the mosquito, *Anopheles gambiae*, to test for the presence of a characteristic metabolite, indole, from harmful coliform bacteria. We demonstrated that recombinantly expressed AgamOBP1 binds indole with high sensitivity. Our proof-of-concept assay is fluorescence-based and demonstrates the usefulness of insect OBPs as detector elements in novel biosensors that rapidly detect the presence of bacterial metabolic markers, and thus of coliform bacteria. We further demonstrated that rAgamOBP1 is suitable for use in portable, inexpensive “dipstick” biosensors that improve upon lateral flow technology since insect OBPs are robust, easily obtainable via recombinant expression, and resist detector “fouling.” Moreover, due to their wide diversity and ligand selectivity, insect chemosensory proteins have other biosensor applications for various analytes. The techniques presented here therefore represent platform technologies applicable to various future devices.

## 1. Introduction

Ensuring water supplies are safe is crucial to public health. Water supply safety concerns are not limited to potable water or even water that is intended for domestic use; irrigation water and water sources not intended for direct human use or consumption must also meet basic safety and hygiene requirements in order to ensure public safety and prevent disease transmission from water-borne microbes. A key indicator organism that reveals contamination in a tested water sample is the bacterium, *Escherichia coli* [1,2]. However, present methods approved by the US Environmental Protection Agency for detecting *E. coli* contamination require at least 24 h [3]. Given the relatively short time required to distribute foods such as vegetables that may have been treated with water, a reliable detection mechanism requiring less time would improve water safety. An interesting characteristic shared by many current contamination detection methods is the essentially retroactive or *ex post facto* nature of their results; however, since point source contamination of water supplies is sometimes swift, a quick, field-deployable device could address water safety concerns on a more immediate basis. Deadly outbreaks of *E. coli* O157:H7, such as the incidents involving spinach grown in California [4,5] and lettuce grown in Arizona [6], highlight the need for a rapid, effective, and cost-efficient *E. coli* detection method, as modern distribution networks can quickly result in contaminated food infections spanning large geographic areas. For example, the 2018 *E. coli* O157:H7 outbreak from Arizona lettuce resulted in 210 cases of infection in 36 states, including 96 hospitalizations and five deaths; one death involved a patient infected across the country in New York [6]. A field-deployable, easy to use biosensor capable of providing rapid results in response to point-source contamination of the water supply would have been particularly valuable in these instances. 

Insects rely on several classes of chemosensory proteins to detect scents and tastes from the environment [7,8] and to regulate crucial responses to environmental stimuli. The odorant-binding proteins (OBPs) are the first chemosensory proteins to bind to an odor or scent molecule from the environment, and are thus responsible for the initial step of molecule recognition. A highly successful and diverse group, insects have evolved a large variety of OBPs that are capable of binding to numerous molecules [7,9,10,11,12,13]. Insect OBPs are relatively small (less than ~20 kDa) proteins assembled with six α-helices and are additionally stabilized by disulfide bridges [14,15,16,17,18,19,20,21,22] to yield a robust and resilient structure. As a result, insect OBPs are characterized by thermal stability and resistance to proteolysis and denaturation as well as the capacity to readily refold upon restoration of favorable conditions [21,23,24,25,26]. Furthermore, many insect species rely on odor detection to regulate crucial behaviors, such as feeding and mating, and this detection of food sources and mates is mediated by insect OBPs and the closely related pheromone-binding proteins (PBPs) respectively [7,8,18,27,28,29,30,31,32,33,34]. Insect OBPs have consequently evolved into a diverse family of proteins with a wide range of analyte specificities and selectivities [28,29,30,31,34,35,36,37,38,39,40,41,42,43,44,45]. These robust, diverse proteins are thus well suited for service as detector elements in novel biosensors [46,47]. 

The advantageous physical and binding properties of insect OBPs [21] have resulted in several attempts at utilizing these proteins in biosensor devices to detect a variety of analytes. OBP14 from the honeybee, *Apis mellifera*, has been used to detect the neuroblastoma biomarker, homovanillic acid, in a device based on reduced graphene oxide (rGO) field-effect transistor (FET) technology [48]. The ligand specificity and selectivity of recombinantly expressed *A. mellifera* OBP14 can be fine-tuned by generating mutant variants; for example, an additional disulfide bridge in the protein’s structure will increase its affinity for a component of plant odors that is also an insect pheromone precursor, eugenol [49]. In fact, the concept of fine-tuning biosensor responses by introducing mutations to an OBP detector element has been explored successfully [50] and may prove useful in future biosensor devices. An rGO-FET OBP14 biosensor can also be used to detect compounds attractive to bees [51]. Likewise, OBP biosensors relying on electrochemical impedance measurements are arguably feasible [52] and current technologies have made the artificial “electronic nose” realistic [53]. These and similar insect OBP-based biosensors demonstrate that proteins derived from the insect chemosensory system are suitable for use as detector elements in biosensors; however, these biosensors are generally complex devices with reporter mechanisms that rely on advanced technologies. 

In contrast, the novel biosensors we have previously described [46,47,54,55] and have now implemented here combine the advantages of an insect OBP-based detector element with simple, established reporter mechanisms based on proven technologies [18,31,34,56,57,58]. Accordingly, we describe two biosensor implementations based on an insect OBP, AgamOBP1 from *Anopheles gambiae*, as the detector element. Both biosensors can detect the characteristic bacterial metabolite, indole [59], quickly and with high sensitivity, making the devices suitable as first-line means of detecting coliform bacterial contamination in water supplies. 

## 2. Materials and Methods

### 2.1. Cloning AgamOBP1

A PCR-amplified DNA fragment encoding AgamOBP1 (AF437884) was cloned into pRSET-B (Thermo Fisher Scientific, Waltham, MA, USA) and soluble recombinant protein (rAgamOBP1) was produced in *E. coli* BL21 Star (DE3) pLysS cells [18,34]. The rAgamOBP1 protein was purified on a nickel-NTA column following the manufacturer’s directions (Thermo Fisher Scientific, Waltham, MA, USA), eluted with 5 mM EDTA and subjected to extensive dialysis against 50 mM Tris-HCl pH 7.4.

### 2.2. Attenu Assay System

**Figure 1:**
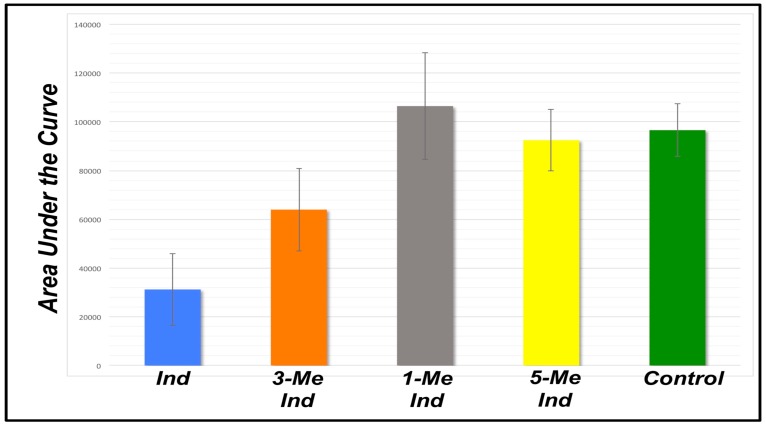
A novel plate-based biosensor to detect indole with high sensitivity. The *Attenu* fluorescence-quenching assay was adapted to develop a biosensor and rAgamOBP1 at 1 μM served as the detector element. The fluorescent dye, 1-NPN, binds rAgamOBP1 and results in a signal that can be detected spectrophotometrically. Analytes capable of binding to rAgamOBP1 displace the dye from the protein’s binding pocket, thus resulting in fluorescence quenching. The tested compounds at 10 μM concentration included indole (Ind), 3-methyl indole (3-Me Ind), 1-methyl indole (1-Me Ind), and 5-methyl indole (5-Me Ind). Six replicates of each compound were evaluated. Three replicates were performed with rAgamOBP1 and buffer alone as controls. The *loss* of signal indicates a binding event; thus, note the signal drop generated by indole and to a lesser degree 3-methyl indole. The bars are STDEV determined with Excel and the raw data are shown in Appendix A Table A1.

**Figure 2:**
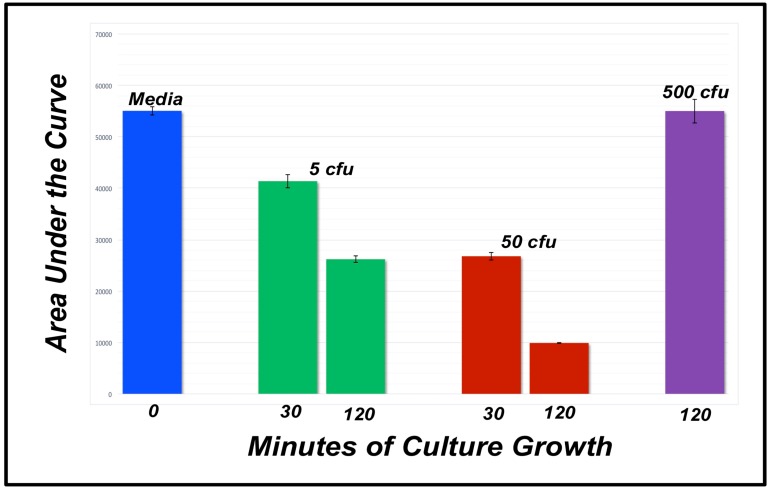
Rapidly detecting *E. coli* cells. The biosensor based on the *Attenu* fluorescence-quenching assay utilized rAgamOBP1 at 1 μM as the detector element. Data were collected using a spectrophotometer. Bar graph of the areas under the response curves for the control (media, no bacteria); 5 cfu of K-12 (green); 50 cfu of K-12 (red); and 500 cfu of an *E. coli* strain (MG1651; Philip Rather, personal communication) without functional tryptophanase and therefore unable to produce indole (purple). The black bars are STDEV plotted with Excel and the raw data are shown in Appendix A Table A2.

**Figure 3:**
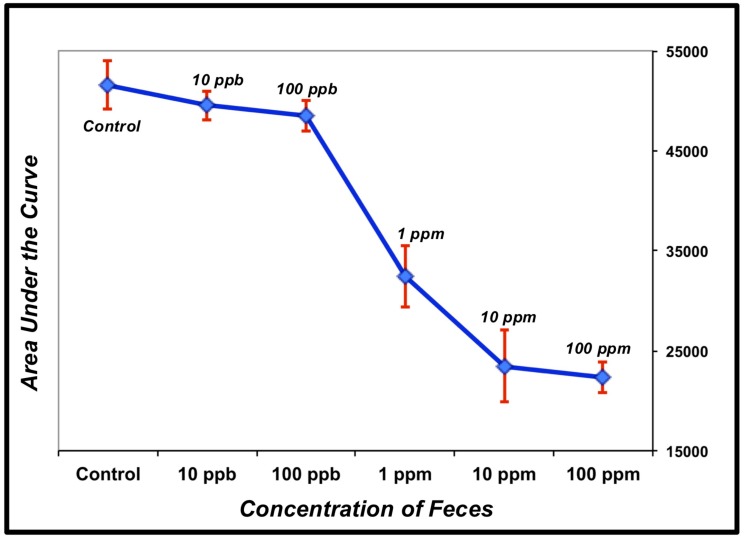
Detecting fecal contamination of water. Data were obtained using the plate-based biosensor with rAgamOBP1 at 1 μM as the detector element. Data were collected using a spectrophotometer. Control samples contain only water. The areas under each curve were plotted on a semi-logarithmic scale in order to evaluate the linearity of the biosensor’s response. The linear range of detection ranges from 10 ppm to 100 ppb. The red bars are STDEV plotted with Excel and the raw data are shown in Appendix A Table A3.

The *Attenu* assay takes advantage of the fluorescent properties of the dye, 1-NPN (N-Phenyl-1-naphthylamine, CAS 90-30-2) [18]. 1-NPN exhibits a detectably altered emission spectrum when interacting with the ligand-binding pocket of insect OBPs in that the peak emission wavelength is shifted from 460 nm to 416 nm and the maximum response amplitude is increased. When a ligand displaces 1-NPN from the OBP’s binding pocket, fluorescent response is reduced. This fluorescence quenching can be detected using a spectrophotometer. The *Attenu* screening system was utilized with concentrations of ligands and rAgamOBP1 in the µM range and fluorescence was detected using a Molecular Devices Gemini XPS spectrofluorometer (Sunnyvale, CA, USA). The results are depicted in Figure 1, Figure 2 and Figure 3.

### 2.3. Lateral Flow Devices

**Figure 4:**
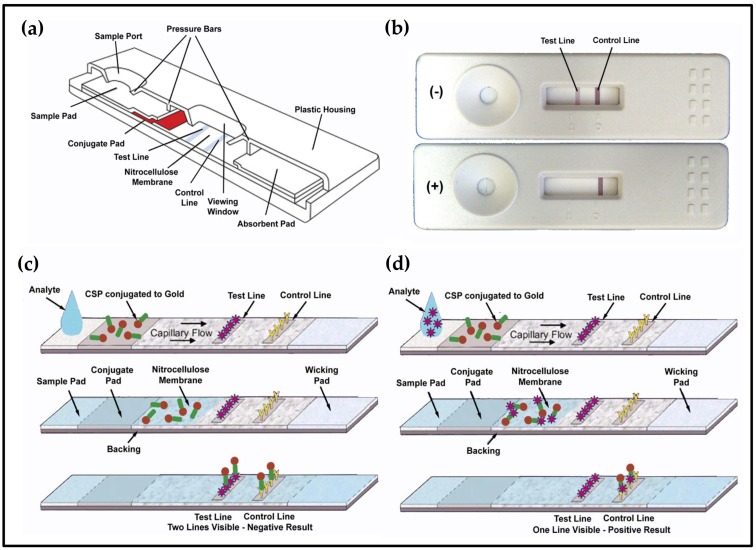
Lateral flow biosensor to detect indole. The detection scheme utilizes rAgamOBP1 that is rendered visible to the naked eye when it is conjugated to colloidal gold. Thus, rAgamOBP1–colloidal gold conjugate lines were striped onto a nitrocellulose membrane sandwiched between sample and conjugate pads. Detection of indole is based on the competition between any indole present in a given sample to be tested and the synthetic ligand in the test line. A *positive* result is reflected by the *loss* of a visible test line on the device and indicates that indole is present in the tested sample. Therefore, the device displays only the control line for a *positive* result. When testing a sample lacking indole, the device displays two lines—the test line and the control line. (**a**) Cut-away diagram of a standard lateral flow device. (**b**) The indole biosensor device. The sample that was added to the upper cassette (-) contained only PBS and shows how a negative result appears (2 lines visible). The sample added to the lower cassette (+) contained PBS plus indole (100 ppm) and demonstrates a positive result (only control line visible). (**c**,**d**) Operating principles of the lateral flow indole biosensor. In (**c**) the control (PBS) result is shown. Since no ligand is present in the analyte, the rAgamOBP1–colloidal gold conjugate is free to bind the test line as well as the control line. A positive result is shown in (**d**). The indole in the analyte binds the rAgamOBP1–colloidal gold conjugate and prevents binding to the test line; therefore, only the control line is visible.

The lateral flow devices utilized absorbent pads supporting a nitrocellulose membrane that is in contact with the sample and conjugate pads. The conjugate pad contains rAgamOBP1 conjugated to 30 nm colloidal gold, which served as a source of color for both test and control lines; colloidal gold is visible to the naked eye. The test line contains a competitive ligand and the control line contains an anti-rAgamOBP1 antibody. If a tested sample contains indole or its derivatives, these molecules will displace the competitive ligand from the test line; a positive result is one in which the visible test line is lost. The control line will verify functionality of the device if a visible signal is produced as the anti-rAgamOBP1 antibodies capture displaced rAgamOBP1. The device is depicted in Figure 4.

Devices are assembled from sheets that are striped with the appropriate molecules using a SynQuad Automated Dispenser (Cartesian Technologies, Irvine, CA, USA) and cut into 5 mm strips. The strips can be supported by an inert, rigid material, such as plastic (e.g., polypropylene).

A tested solution travels along the strip via capillary action with the speed of flow determined by the pore size of the membrane. Visible results appear in less than 20 min. More detailed analyses of the results can be achieved if the strips are allowed to dry for 24 h and then read using a Qiagen ESE-Quant GOLD scanner (Qiagen, Hilden, Germany) to determine the absorbance by position in 40 μm increments. The scanner merely allows for quantification as well as detection of differences not obvious to the naked eye; it is not necessary for operation of the device or rapid detection of indole.

In order to generate a test strip capable of acting as a real-time positive control, we used the *Attenu* assay [18] to isolate a synthetic ligand for rAgamOBP1 from a combinatorial chemical library (ChemBridge, San Diego, CA, USA). The synthetic ligand was used in the positive control strip. Tests were performed using a 2X ligand dilution series in which strips are exposed to differing concentrations of indolepropionic acid bound to BSA (IPA-BSA) with constant amounts of Hi-Veg media (containing high levels of tryptophan, the metabolic breakdown of which produces indole) and of rAgamOBP1 conjugated to 30 nm colloidal gold.

The completed device was evaluated using *E. coli* K-12 cells (ATCC, Manassas, VA, USA). Liquid cultures of K-12 were diluted 1000X in Hi-Veg media to provide a source of tryptophan that the cells metabolize into indole. Cells were incubated for 60 min and 500 μL aliquots were removed every 30 min for testing. Tests lasted 3 h and the absorbance at OD_600_ of tested samples ranged from 0.005 A to 0.05 A.

## 3. Results

### 3.1. Fluorescence-Quenching Assay to Detect Coliform Bacteria and Fecal Contamination

The *Attenu* assay system [18] was used as the platform for a plate-based biosensor with recombinant AgamOBP1 at 1 μM as the detector element. This device was tested with samples of bacterial cells at various concentrations as well as aqueous solutions containing indole.

**Figure A1:**
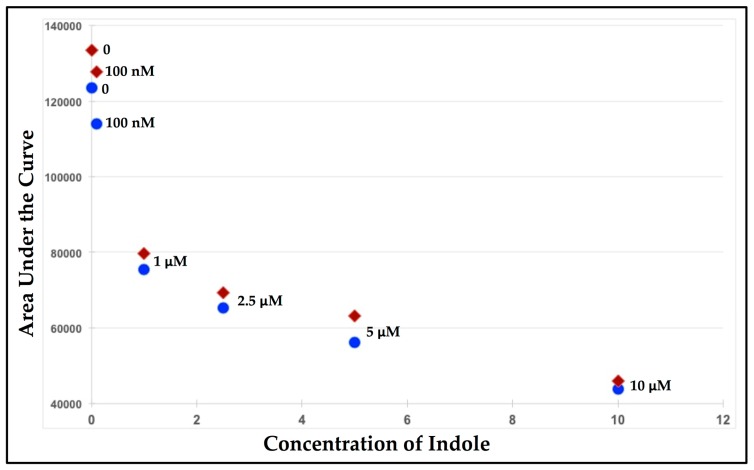
Response of AgamOBP1 to various dilutions of indole using the Attenu assay. Concentrations of indole including, from left to right, 0, 100 nM, 1 μM, 2.5 μM, 5 μM, and 10 μM were tested with the Attenu assay. Shown on the plot are two separate experiments run with the same indole dilutions. The first experiment is shown as blue circles, the second as red diamonds. Note that a drop in response is visible even at 100 nM.

Indole detection testing was performed with varying concentrations of indole in water. Initial results indicated that the biosensor detected indole at concentrations less than 100 nM (~5 ppb; Appendix A Figure A1) and detection requires 30 min or less, making this biosensor not only more sensitive than the standard method of detection but also much more rapid [3]. Expanded testing using indole derivatives including 1-methyl indole, 3-methyl indole (skatole), and 5-methyl indole each at 10 μM concentrations showed binding to skatole only (Figure 1).

### 3.2. Detecting E. coli Cells

*E. coli* detection was tested for response time and sensitivity. The plate-based biosensor detected 50 cfu (colony forming units) of *E. coli* strain K-12 cells after a 30-min incubation, and the signal became increasingly robust up to the maximum incubation time of 120 min (Figure 2). The plate-based biosensor was also tested for sensitivity, and detected less than 5 colony-forming units (cfu) of *E. coli* after a 30-min incubation (Figure 2). 

### 3.3. Detecting Fecal Contamination

The plate-based biosensor was also tested for the ability to detect fecal contamination in an aqueous solution. Canine feces were diluted in water at abundances ranging from 100 ppm (parts per million) to 10 ppb (parts per billion). The biosensor was effective for the detection of fecal contamination in water with a reliable detection limit of 100 ppb. The linear range of detection is from 10 ppm to 100 ppb (Figure 3), making this biosensor a responsive, sensitive instrument for fecal contamination of water supplies.

### 3.4. Low-Cost Portable Lateral Flow Device to Detect Fecal Contamination

Lateral flow devices were assembled in a format resembling commercially available pregnancy test kits with blank “cassettes” made of inert plastic (e.g., polypropylene). The device is based on competitive binding between an analyte in the tested sample and a synthetic ligand to rAgamOBP1 and results are interpreted based on the color of a test line and a control line. The device detected indole in aqueous solutions at concentrations as low as 100 ppm (Figure 4).

## 4. Discussion

**Figure A2:**
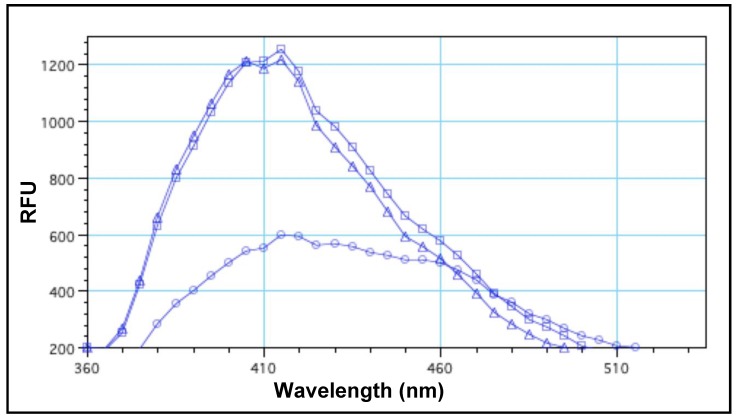
rAgamOBP1 activity after 5 years of storage. 10 μM rAgamOBP1 in 50 mM Tris-HCl pH 7.4 tested for response in the *Attenu* assay system with the fluorescent dye, 1-NPN, that binds to the protein and results in a signal that is detectable spectrophotometrically. Data shown as collected directly from the Gemini XPS spectrofluorometer (Molecular Devices, Sunnyvale, CA, USA). *x*-axis: wavelength in nm; *y*-axis: response units. Open circle: control (5 μM 1-NPN in 50 mM Tris-HCl pH 7.4); open square: freshly-purified 10 μM rAgamOBP1 with 5 μM 1-NPN in 50 mM Tris-HCl pH 7.4; open triangle: stored 10 μM rAgamOBP1 with 5 μM 1-NPN in 50 mM Tris-HCl pH 7.4. The rAgamOBP1 had been stored in 50 mM Tris-HCl pH 7.4 for 5 years.

Herein we describe a novel sensor technology encompassing an olfactory protein from *Anopheles gambiae* mosquitoes, AgamOBP1, as the detector element [7,46,47,54,55,60]. AgamOBP1 binds analytes associated with coliform bacteria and does so with high specificity and sensitivity [18,34], allowing the rapid detection of low level *E. coli* contamination in water supplies. Moreover, AgamOBP1 is a resilient protein that has the typically robust OBP structure with six α-helices that are stabilized by disulfide bridges [14,15,16,17,18,19,20,21,22]; in our laboratory, samples of AgamOBP1 remained active after being stored in 50 mM Tris-HCl pH 7.4 for up to 5 years (Appendix A, Figure A2). We demonstrate two implementations of AgamOBP1 as a detector element in biosensors that indicate the presence of coliform bacterial metabolites in aqueous solutions [46].

One implementation of an OBP-based biosensor is based on an established reporter mechanism, the *Attenu* assay [18,31,34,46,61], and can be implemented in vitro (Figure 1, Figure 2 and Figure 3). In this implementation, based on competitive binding between either a fluorescent indicator dye or any given analyte [21,62] for AgamOBP1’s binding pocket [18,34], recombinantly expressed AgamOBP1 can detect the bacterial metabolite, indole [59], in concentrations below 1 μM in aqueous solutions (Appendix A Figure A1). The plate-based biosensor can also detect *E. coli* cells rapidly, requiring less than 30 min for a result (Figure 2).

However, insect OBP-based biosensors can also be incorporated into lateral flow devices [46] assembled as nitrocellulose strips or sheets with a paper sample pad or wick and supported on a plastic (e.g., PVC) substrate [56,58,63,64,65,66]. Such devices are similar in implementation to commonly available pregnancy tests or “dipsticks”, and can take advantage of existing packaging facilities for mass production purposes as a result [56,57,58]. In this biosensor, a test strip contains a known ligand for AgamOBP1, and a control strip contains an antibody against AgamOBP1. When an aqueous analyte mixture is introduced, an AgamOBP1–colloidal gold complex travels laterally along the device’s surface. If AgamOBP1 has bound none of the ligands from the tested aqueous solution, then it will bind the test strip, deposit colloidal gold, and cause a color change. A ligand from the tested solution bound by AgamOBP1 will prevent this color change in the test strip; thus, the *absence* of a color change on the test strip indicates a positive result. The control strip verifies that AgamOBP1 is present and travelled laterally along the device.

Both implementations of insect OBP-based biosensors yield data in less than 1 h. Our results indicate the OBP-based approach is at least one thousand times (1000X) more sensitive than previous methods (e.g., the indole spot test) [59] used for determining the presence of indole. The devices presented herein thus provide proof of concept for insect OBP-based biosensors in general [46,47,54,55]. Future OBP-based biosensors can take advantage of the high sensitivity and versatility of the insect chemosensory system [7,9,10,11,12,13] to detect a wide variety of analytes.

The techniques described here can be used to assemble OBP-based biosensors for a wide variety of applications including the detection of environmental, chemical, or biological compounds or contaminants [46,47,54,55]. Such uses include the detection of toxins or stereoisomers generated during chemical or pharmaceutical synthesis [21,67,68,69], the detection of harmful volatile organic compounds (VOCs), quality control of foods and pharmaceuticals [21,70,71], and the detection of volatile compounds present in weapons or explosives [21,72,73]. These biosensors can also be used in medical diagnostics [21,74,75,76,77,78,79,80,81,82,83,84,85,86,87,88] as well as numerous other applications where high detector stability, high sensitivity, and analyte selectivity are required. When used as detectors of coliform bacteria in aqueous solutions, the OBP-based biosensors described have specific advantages over bacterial culture- or plate-based detection methods in that the latter can only reveal the presence of living cells and require up to 24 h to do so. OBP-based detectors are not only capable of rapid detection but can also be targeted against coliform-specific metabolic byproducts—that is, the biosensors detect coliform contamination whether the sample contains living cells or not [46,47,54,55]. Unlike PCR-based methods, OBP-based biosensors can be implemented as simple devices that do not require high levels of end-user expertise [56,58,63,64,65,66]. Furthermore, although antibody-based biosensors are established [89,90,91], they are limited to detecting analytes that are sufficiently antigenic. Since OBP-based detector elements do not rely on antibodies, they can detect analytes with poor antigenic properties. Thus, the described platform technology has immediate application to a variety of important sensor and detector implementations.

## 5. Patents

Woods, D.F.; Dimitratos, S.D.; Justice, R.W. Methods to Utilize Invertebrate Chemosensory Proteins for Industrial and Commercial Uses. CA 2666662, 31 July 2008, 2013.

Woods, D.F.; Dimitratos, S.D.; Justice, R.W. Methods to Utilize Invertebrate Chemosensory Proteins for Industrial and Commercial Uses. AU 2007345342, 14 May 2009, 2016.

Woods, D.F.; Dimitratos, S.D.; Justice, R.W. Methods to Utilize Invertebrate Chemosensory Proteins for Industrial and Commercial Uses. EP 2087351, 12 August 2009, 2012.

Woods, D.F.; Dimitratos, S.D.; Justice, R.W. Methods to Utilize Invertebrate Chemosensory Proteins for Industrial and Commercial Uses. NZ 576264, 31 March 2012, 2012.

## Appendix A

**Table A1:** Raw numbers and statistical analysis of the data shown in Figure 1. Area under the response curve from the *Attenu* assay is shown in the first 6 rows. The indole derivative is listed on top. Averages and statistical analysis are shown in the last 3 rows.

	Indole	3-Methyl Indole	1-Methyl Indole	5-Methyl Indole	Buffer
	15568	41586	125719	105668	106916
	33036	47193	126569	77743	85425
	10714	73470	99218	83534	97430
	41302	81037	67384	104760	--
	39975	79938	111689	82392	--
	46871	60676	108177	100800	--
AVG	**31244**	**63983**	**106459**	**92483**	**96590**
STDEV	±14779	±16911	±21840	±12593	±10770
*t*-test	**−9.89**	**−4.31**	**1.01**	**−0.73**	**--**

**Table A2:** Raw numbers and statistical analysis of the data shown in Figure 2. Area under the response curve from the *Attenu* assay is shown in the first 3 rows. The type of bacteria is listed above the Table. Averages and statistical analysis are shown in the last 3 rows.

	Media	5 cfu 30 mins	5 cfu 60 mins	50 cfu 30 mins	50 cfu 60 mins	500 cfu 120 mins
	54011	43275	28041	25901	9962	55488
	58867	42728	26925	30529	9852	57053
	52270	38129	23716	22961	9907	52489
AVG	**55049**	**42044**	**26227**	**26463**	**9907**	**55010**
STDEV	±3418	±1680	±2245	±3815	±1028	±2319
*t*-test	**--**	**−8.38**	**−22.23**	**−12.98**	**−76.01**	**−0.03**

**Table A3:** Raw numbers and statistical analysis of the data shown in Figure 3. Area under the response curve from replicates in the *Attenu* assay is shown in the first 3 rows. The concentration of feces tested is listed on top. Averages and statistical analysis are shown in the last 3 rows.

	Buffer	10 ppb	100 ppb	1 ppm	10 ppm	100 ppm
	53596	50925	48663	35905	27464	22986
	52349	48016	50002	30033	22567	23465
	48890	49705	46901	31285	20376	20567
AVG	**51611**	**49548**	**48522**	**32407**	**23469**	**22339**
STDEV	±2438	±1460	±1555	±3092	±3629	±1553
*t*-test	**--**	**−3.16**	**−4.44**	**−13.90**	**−17.34**	**−42.13**

## References

[1] Todar K. (2005). Ken Todar’s Microbial World: E. coli Infections.

[2] Cakir I., Dogan H.B., Baspinar E., Keven F., Halkman A.K. (2002). The need for confirmation in coliform and E. coli enumeration in foods. Turk. J. Vet. Anim. Sci..

[3] EPA (2003). Guidelines for Establishing Test Procedures for the Analysis of Pollutants. Analytical Methods for Biological Pollutants in Ambient Water.

[4] CDC (2006). Ongoing multistate outbreak of Escherichia coli serotype O157:H7 infections associated with consumption of fresh spinach—United States, September 2006. MMWR Morb. Mortal. Wkly. Rep..

[5] Charatan F. (2006). FDA warns US consumers not to eat spinach after E coli outbreak. BMJ.

[6] CDC Multistate Outbreak of *E. coli* O157:H7 Infections Linked to Romaine Lettuce. https://www.cdc.gov/ecoli/2018/o157h7-04-18/.

[7] Justice R.W., Biessmann H., Walter M.F., Dimitratos S.D., Woods D.F. (2003). Genomics spawns novel approaches to mosquito control. Bioessays.

[8] Justice R.W., Dimitratos S., Walter M.F., Woods D.F., Biessmann H. (2003). Sexual dimorphic expression of putative antennal carrier protein genes in the malaria vector Anopheles gambiae. Insect Mol. Biol..

[9] Field L.M., Pickett J.A., Wadhams L.J. (2000). Molecular studies in insect olfaction. Insect Mol. Biol..

[10] Krieger J., Breer H. (1999). Olfactory reception in invertebrates. Science.

[11] Takken W. (1991). The role of olfaction in host- seeking of mosquitoes: A review. Insect Sci. Appl..

[12] Briand L., Nespoulous C., Huet J.C., Takahashi M., Pernollet J.C. (2001). Ligand binding and physico-chemical properties of ASP2, a recombinant odorant-binding protein from honeybee (*Apis mellifera* L.). Eur. J. Biochem..

[13] Calvello M., Brandazza A., Navarrini A., Dani F.R., Turillazzi S., Felicioli A., Pelosi P. (2005). Expression of odorant-binding proteins and chemosensory proteins in some Hymenoptera. Ins. Biochem. Mol. Biol..

[14] Scaloni A., Monti M., Angeli S., Pelosi P. (1999). Structural analysis and disulfide-bridge pairing of two odorant-binding proteins from Bombyx mori. Biochem. Biophys. Res. Commun..

[15] Briand L., Nespoulous C., Huet J.C., Pernollet J.C. (2001). Disulfide pairing and secondary structure of ASP1, an olfactory-binding protein from honeybee (*Apis mellifera* L.). J. Pept. Res..

[16] Wogulis M., Morgan T., Ishida Y., Leal W.S., Wilson D.K. (2006). The crystal structure of an odorant binding protein from Anopheles gambiae: Evidence for a common ligand release mechanism. Biochem. Biophys. Res. Commun..

[17] Lagarde A., Spinelli S., Tegoni M., He X., Field L., Zhou J.J., Cambillau C. (2011). The crystal structure of odorant binding protein 7 from Anopheles gambiae exhibits an outstanding adaptability of its binding site. J. Mol. Biol..

[18] Rusconi B., Maranhao A.C., Fuhrer J.P., Krotee P., Choi S.H., Grun F., Thireou T., Dimitratos S.D., Woods D.F., Marinotti O. (2012). Mapping the Anopheles gambiae Odorant Binding Protein 1 (AgamOBP1) using modeling techniques, site directed mutagenesis, circular dichroism and ligand binding assays. Biochim. Biophys. Acta.

[19] Tsitsanou K.E., Thireou T., Drakou C.E., Koussis K., Keramioti M.V., Leonidas D.D., Eliopoulos E., Iatrou K., Zographos S.E. (2012). Anopheles gambiae odorant binding protein crystal complex with the synthetic repellent DEET: Implications for structure-based design of novel mosquito repellents. Cell. Mol. Life Sci..

[20] Spinelli S., Lagarde A., Iovinella I., Legrand P., Tegoni M., Pelosi P., Cambillau C. (2012). Crystal structure of Apis mellifera OBP14, a C-minus odorant-binding protein, and its complexes with odorant molecules. Insect Biochem. Mol. Biol..

[21] Pelosi P., Mastrogiacomo R., Iovinella I., Tuccori E., Persaud K.C. (2014). Structure and biotechnological applications of odorant-binding proteins. Appl. Microbiol. Biotechnol..

[22] Leal W.S., Nikonova L., Peng G. (1999). Disulfide structure of the pheromone binding protein from the silkworm moth, Bombyx mori. FEBS Lett..

[23] Ban L., Scaloni A., Brandazza A., Angeli S., Zhang L., Yan Y., Pelosi P. (2003). Chemosensory proteins of *Locusta migratoria*. Insect Mol. Biol..

[24] Ban L., Scaloni A., D’Ambrosio C., Zhang L., Yahn Y., Pelosi P. (2003). Biochemical characterization and bacterial expression of an odorant-binding protein from *Locusta migratoria*. Cell. Mol. Life Sci..

[25] Schwaighofer A., Kotlowski C., Araman C., Chu N., Mastrogiacomo R., Becker C., Pelosi P., Knoll W., Larisika M., Nowak C. (2014). Honey bee odorant-binding protein 14: Effects on thermal stability upon odorant binding revealed by FT-IR spectroscopy and CD measurements. Eur. Biophys. J..

[26] Calvello M., Guerra N., Brandazza A., D’Ambrosio C., Scaloni A., Dani F.R., Turillazzi S., Pelosi P. (2003). Soluble proteins of chemical communication in the social wasp *Polistes dominulus*. Cell. Mol. Life Sci..

[27] Bohbot J., Vogt R.G. (2005). Antennal expressed genes of the yellow fever mosquito (*Aedes aegypti* L.); characterization of odorant-binding protein 10 and takeout. Insect Biochem. Mol. Biol..

[28] Deng Y., Yan H., Gu J., Xu J., Wu K., Tu Z., James A.A., Chen X. (2013). Molecular and functional characterization of odorant-binding protein genes in an invasive vector mosquito, Aedes albopictus. PLoS ONE.

[29] Harada E., Nakagawa J., Asano T., Taoka M., Sorimachi H., Ito Y., Aigaki T., Matsuo T. (2012). Functional Evolution of Duplicated Odorant-Binding Protein Genes, *Obp57d* and *Obp57e*, in *Drosophila*. PLoS ONE.

[30] Matsuo T., Sugaya S., Yasukawa J., Aigaki T., Fuyama Y. (2007). Odorant-binding proteins OBP57d and OBP57e affect taste perception and host-plant preference in *Drosophila sechellia*. PLoS Biol..

[31] Patt J.M., Woods D., Dimitratos S., Meikle W.G., Stockton D., Lapointe S.L., Mafra-Neto A., Beck J., Coats J., Duke S.O., Koivunen M. (2013). Novel synthetic ligands enhance the behavioral responses of Asian citrus psyllid to naturally occurring host-plant volatiles. Pest Management with Natural Products.

[32] Swarup S., Williams T.I., Anholt R.R. (2011). Functional dissection of Odorant binding protein genes in Drosophila melanogaster. Genes Brain Behav..

[33] Vosshall L.B., Stensmyr M.C. (2005). Wake up and smell the pheromones. Neuron.

[34] Biessmann H., Andronopoulou E., Biessmann M.R., Douris V., Dimitratos S.D., Eliopoulos E., Guerin P.M., Iatrou K., Justice R.W., Krober T. (2010). The Anopheles gambiae odorant binding protein 1 (AgamOBP1) mediates indole recognition in the antennae of female mosquitoes. PLoS ONE.

[35] Bohbot J., Sobrio F., Lucas P., Nagnan-Le Meillour P. (1998). Functional characterization of a new class of odorant-binding proteins in the moth *Mamestra brassicae*. Biochem. Biophys. Res. Commun..

[36] Danty E., Briand L., Michard-Vanhee C., Perez V., Arnold G., Gaudemer O., Huet D., Huet J.C., Ouali C., Masson C. (1999). Cloning and expression of a queen pheromone-binding protein in the honeybee: An olfactory-specific, developmentally regulated protein. J. Neurosci..

[37] Davrazou F., Dong E., Murphy E.J., Johnson H.T., Jones D.N. (2011). New insights into the mechanism of odorant detection by the malaria—Transmitting mosquito anopheles gambiae. J. Biol. Chem..

[38] Guo H., Huang L.Q., Pelosi P., Wang C.Z. (2012). Three pheromone-binding proteins help segregation between two Helicoverpa species utilizing the same pheromone components. Insect Biochem. Mol. Biol..

[39] He X., Tzotzos G., Woodcock C., Pickett J.A., Hooper T., Field L.M., Zhou J.J. (2010). Binding of the general odorant binding protein of Bombyx mori BmorGOBP2 to the moth sex pheromone components. J. Chem. Ecol..

[40] Hekmat-Scafe D.S., Dorit R.L., Carlson J.R. (2000). Molecular evolution of odorant-binding protein genes OS-E and OS-F in Drosophila. Genetics.

[41] Leal W.S. (2013). Odorant reception in insects: Roles of receptors, binding proteins, and degrading enzymes. Annu. Rev. Entomol..

[42] Liu S.J., Liu N.Y., He P., Li Z.Q., Dong S.L., Mu L.F. (2012). Molecular characterization, expression patterns, and ligand-binding properties of two odorant-binding protein genes from *Orthaga achatina* (Butler) (Lepidoptera: Pyralidae). Arch. Insect Biochem. Physiol..

[43] Matsuo T. (2008). Rapid evolution of two odorant-binding protein genes, Obp57d and Obp57e, in the Drosophila melanogaster species group. Genetics.

[44] Murphy E.J., Booth J.C., Davrazou F., Port A.M., Jones D.N. (2013). Interactions of Anopheles gambiae odorant binding proteins with a human-derived repellent: Implications for the mode of action of DEET. J. Biol. Chem..

[45] Pelletier J., Guidolin A., Syed Z., Cornel A.J., Leal W.S. (2010). Knockdown of a mosquito odorant-binding protein involved in the sensitive detection of oviposition attractants. J. Chem. Ecol..

[46] Woods D.F., Dimitratos S.D., Justice R.W. (2009). Methods to Utilize Invertebrate Chemosensory Proteins for Industrial and Commercial Uses. Patent No..

[47] Woods D.F., Dimitratos S.D., Justice R.W. (2009). Methods to Utilize Invertebrate Chemosensory Proteins for Industrial and Commercial Uses. Patent No..

[48] Reiner-Rozman C., Kotlowski C., Knoll W. (2016). Electronic Biosensing with Functionalized rGO FETs. Biosensors.

[49] Kotlowski C., Larisika M., Guerin P.M., Kleber C., Kröber T., Mastrogiacomo R., Nowak C., Pelosi P., Schütz S., Schwaighofer A. (2018). Fine discrimination of volatile compounds by graphene-immobilized odorant-binding proteins. Sens. Actuators B Chem..

[50] Yi X., Zhang Y., Wang P., Qi J., Hu M., Zhong G. (2015). Ligands binding and molecular simulation: The potential investigation of a biosensor based on an insect odorant binding protein. Int. J. Biol. Sci..

[51] Larisika M., Kotlowski C., Steininger C., Mastrogiacomo R., Pelosi P., Schutz S., Peteu S.F., Kleber C., Reiner-Rozman C., Nowak C. (2015). Electronic Olfactory Sensor Based on A. mellifera Odorant-Binding Protein 14 on a Reduced Graphene Oxide Field-Effect Transistor. Angew. Chem. Int. Ed. Engl..

[52] Lu Y., Li H., Zhuang S., Zhang D., Zhang Q., Zhou J., Dong S., Liu Q., Wang P. (2014). Olfactory biosensor using odorant-binding proteins from honeybee: Ligands of floral odors and pheromones detection by electrochemical impedance. Sens. Actuators B Chem..

[53] Dung T.T., Oh Y., Choi S.J., Kim I.D., Oh M.K., Kim M. (2018). Applications and Advances in Bioelectronic Noses for Odour Sensing. Sensors.

[54] Woods D.F., Dimitratos S.D., Justice R.W. (2012). Methods to Utilize Invertebrate Chemosensory Proteins for Industrial and Commercial Uses. Patent.

[55] Woods D.F., Dimitratos S.D., Justice R.W. (2008). Methods to Utilize Invertebrate Chemosensory Proteins for Industrial and Commercial Uses. Patent.

[56] O’Farrell B., Konrad K.D. (2010). Diagnostic Consulting Netowrk: On the Market and Intellectual Property Landscape of Lateral Flow Devices.

[57] Park J., Shin J.H., Park J.K. (2016). Pressed Paper-Based Dipstick for Detection of Foodborne Pathogens with Multistep Reactions. Anal. Chem..

[58] Shin J.H., Hong J., Go H., Park J., Kong M., Ryu S., Kim K.P., Roh E., Park J.K. (2018). Multiplexed Detection of Foodborne Pathogens from Contaminated Lettuces Using a Handheld Multistep Lateral Flow Assay Device. J. Agric. Food Chem..

[59] Lombard G.L., Dowell V.R. (1983). Comparison of three reagents for detecting indole production by anaerobic bacteria in microtest systems. J. Clin. Microbiol..

[60] Biessmann H., Walter M.F., Dimitratos S., Woods D. (2002). Isolation of cDNA clones encoding putative odourant binding proteins from the antennae of the malaria-transmitting mosquito, Anopheles gambiae. Insect Mol. Biol..

[61] Dimitratos S., Justice R., Woods D.F. (2011). Development of Novel Attractants for the Asian Citrus Psyllid, *Diaphorina citri* Kuwayama. Citrograph.

[62] Mastrogiacomo R., Iovinella I., Napolitano E. (2014). New fluorescent probes for ligand-binding assays of odorant-binding proteins. Biochem. Biophys. Res. Commun..

[63] Rao R.S., Ablala J.S., Lane S.L., Matthews D.L., Fisher A.M., Lambert J.L., Coleman M.A. (2005). Developing rapid, point-of-care, multiplex detection for use in lateral flow devices. Proc. SPIE.

[64] Liu J., Mazumdar D., Lu Y. (2006). A Simple and Sensitive “Dipstick” Test in Serum Based on Lateral Flow Separation of Adaptamer-Linked Nanostructures. Angew. Chem. Int. Ed. Engl..

[65] Rosen S. (2007). Lateral Flow Technology and the Future of Point of Care Diagnostics.

[66] Dewey F.M., Steel C.C., Gurr S.J. (2013). Lateral-flow devices to rapidly determine levels of stable Botrytis antigens in table and dessert wines. Am. J. Enol. Vitic..

[67] Backman A.C., Anderson P., Bengtsson M., Lofqvist J., Unelius C.R., Witzgall P. (2000). Antennal response of codling moth males, *Cydia pomonella* L. (Lepidoptera: Tortricidae), to the geometric isomers of codlemone and codlemone acetate. J. Comp. Physiol. A.

[68] Plettner E., Lazar J., Prestwich E.G., Prestwich G.D. (2000). Discrimination of pheromone enantiomers by two pheromone binding proteins from the gypsy moth Lymantria dispar. Biochemistry.

[69] Wojtasek H., Hansson B.S., Leal W.S. (1998). Attracted or repelled?—A matter of two neurons, one pheromone binding protein, and a chiral center. Biochem. Biophys. Res. Commun..

[70] Ouellette J. (1999). Electronic noses sniff out new markets. Ind. Phys..

[71] Canhoto O.F., Magan N. (2003). Potential for detection of microorganisms and heavy metals in potable water using electronic nose technology. Biosens. Bioelectron..

[72] Rains G.C., Utley S.L., Lewis W.J. (2005). Behavioral monitoring of trained insects for chemical detection. Biotechnol. Prog..

[73] Rodacy P.J., Bender S., Bromenshenk J., Henderson C., Bender G. (2002). Deployment of honeybees to detect explosives and other agents of harm. Proc. SPIE.

[74] Di Natale C., Macagnano A., Martinelli E., Paolesse R., D’Arcangelo G., Roscioni C., Finazzi-Agro A., D’Amico A. (2003). Lung cancer identification by the analysis of breath by means of an array of non-selective gas sensors. Biosens. Bioelectron..

[75] Pavlou A.K., Turner A.P. (2000). Sniffing out the truth: Clinical diagnosis using the electronic nose. Clin. Chem. Lab. Med..

[76] Yu F., Persson B., Lofas S., Knoll W. (2004). Surface plasmon fluorescence immunoassay of free prostate-specific antigen in human plasma at the femtomolar level. Anal. Chem..

[77] Fradet Y., Saad F., Aprikian A., Dessureault J., Elhilali M., Trudel C., Masse B., Piche L., Chypre C. (2004). uPM3, a new molecular urine test for the detection of prostate cancer. Urology.

[78] Saad F. (2005). UPM3: Review of a new molecular diagnostic urine test for prostate cancer. Can. J. Urol..

[79] Mutlu N., Turkeri L., Emerk K. (2003). Analytical and clinical evaluation of a new urinary tumor marker: Bladder tumor fibronectin in diagnosis and follow-up of bladder cancer. Clin. Chem. Lab. Med..

[80] Phillips M., Cataneo R.N., Condos R., Ring Erickson G.A., Greenberg J., La Bombardi V. (2004). Volatile markers of pulmonary tuberculosis in the breath. Eur. Respir. J..

[81] Phillips M., Cataneo R.N., Ditkoff B.A., Fisher P., Greenberg J., Gunawardena R., Kwon C.S., Rahbari-Oskoui F., Wong C. (2003). Volatile markers of breast cancer in the breath. Breast J..

[82] Phillips M., Gleeson K., Hughes J.M., Greenberg J., Cataneo R.N., Baker L., McVay W.P. (1999). Volatile organic compounds in breath as markers of lung cancer: A cross-sectional study. Lancet.

[83] Phillips M., Cataneo R.N., Cummin A.R., Gagliardi A.J., Gleeson K., Greenberg J., Maxfield R.A., Rom W.N. (2003). Detection of lung cancer with volatile markers in the breath. Chest.

[84] Pickel D., Manucy G.P., Walker D.B., Hall S.B., Walker J.C. (2004). Evidence for canine olfactory detection of melanoma. Appl. Anim. Behav. Sci..

[85] Dalton P., Gelperin A., Preti G. (2004). Volatile metabolic monitoring of glycemic status in diabetes using electronic olfaction. Diabetes Technol. Ther..

[86] Sieg A., Guy R.H., Delgado-Charro M.B. (2005). Noninvasive and minimally invasive methods for transdermal glucose monitoring. Diabetes Technol. Ther..

[87] Phillips M., Cataneo R.N., Cheema T., Greenberg J. (2004). Increased breath biomarkers of oxidative stress in diabetes mellitus. Clin. Chim. Acta.

[88] Newman J.D., Turner A.P. (2005). Home blood glucose biosensors: A commercial perspective. Biosens. Bioelectron..

[89] Haasnoot W., Smits N.G., Kemmers-Voncken A.E., Bremer M.G. (2004). Fast biosensor immunoassays for the detection of cows’ milk in the milk of ewes and goats. J. Dairy Res..

[90] Vikholm-Lundin I., Albers W.M. (2006). Site-directed immobilisation of antibody fragments for detection of C-reactive protein. Biosens. Bioelectron..

[91] Verma N., Bhardwaj A. (2015). Biosensor Technology for Pesticides—A Review. Appl. Biochem. Biotechnol..

